# Supplementary material for: A Likelihood Approach for Real-Time Calibration of Stochastic Compartmental Epidemic Models
Source: PLoS Comput Biol. 2017 Jan 17;13(1):e1005257. doi: 10.1371/journal.pcbi.1005257 (PMC5240920; doi:10.1371/journal.pcbi.1005257)
Supplement: S1 File — (TAR.GZ) [file pcbi.1005257.s014.tar.gz › HSPH_Online-SI-Revision/EnKF07/n10-extreme/n10-extreme_EnKF_table8.pdf]

1	R0	{2.68692, 12.5884}	{2.11469, 4.87169}	{{0.780591, 3.5582}, {3., 6.54342}}	{35.2795, 61.3001}	inf	138.	431.698	{134.765, 1190.27}	216.308	0.311732	newly	61.	568.19	{92., 1788.}	831.525	0.652482	Reff	0.251496	0.424334	{0.132087, 0.797837}	97.2308	ar	9089.	16 883.8	{15 632., 20 450.}	85.7606
2	R0	{1.79802, 12.5412}	{2.01417, 5.26728}	{{0.458999, 1.23827}, {3., 8.63221}}	{69.5359, 58.0002}	inf	348.	716.241	{227.67, 227.67}	176.667	0.32409	newly	172.	540.05	{124., 477.}	234.692	0.316517	Reff	0.525561	0.405792	{0.0581625, 0.759757}	42.2866	ar	7412.	16 058.1	{14 640., 15 717.}	116.65
3	R0	{2.6174, 10.1105}	{2.53487, 5.12665}	{{1.89254, 2.51954}, {3., 7.9979}}	{20.1097, 50.847}	inf	48.	69.9757	{18.7096, 65.2204}	76.415	0.183764	newly	29.	111.04	{13., 142.}	302.138	0.433885	Reff	0.238183	0.651108	{0.15346, 1.89235}	189.972	ar	9109.	17 182.5	{15 748., 23 573.}	88.6318
4	R0	{2.27532, 14.3149}	{1.94783, 7.07136}	{{0.427729, 3.60883}, {3., 10.9759}}	{51.9757, 50.6014}	inf	238.	840.858	{352.439, 1232.53}	260.31	0.457115	newly	95.	922.93	{237., 1668.}	878.474	0.770774	Reff	0.382254	0.401732	{0.113362, 0.653109}	69.541	ar	8478.	16 901.9	{15 408., 20 876.}	99.3613
5	R0	{2.96649, 18.0674}	{2.12781, 4.92224}	{{0.613188, 1.49018}, {3., 8.09136}}	{29.5371, 72.7562}	inf	369.	228.267	{94.5554, 170.948}	87.0702	0.533063	newly	132.	695.41	{68., 1644.}	477.008	0.360946	Reff	0.177989	0.442635	{0.0843462, 0.684608}	180.986	ar	9476.	15 165.1	{14 191., 16 394.}	60.0365
6	R0	{2.94778, 16.0002}	{2.17192, 5.17693}	{{0.87275, 3.51985}, {3., 6.53273}}	{28.4815, 67.6446}	inf	276.	252.38	{114.971, 481.039}	87.2191	0.432574	newly	93.	315.06	{72., 578.}	255.677	0.29987	Reff	0.21961	0.399807	{0.113992, 0.630866}	107.662	ar	9321.	15 976.2	{14 940., 19 965.}	71.4001
7	R0	{2.17562, 11.7184}	{2.67236, 7.87435}	{{2.13301, 0.0605911}, {3., 13.6463}}	{23.947, 51.4436}	inf	199.	609.95	{15.7897, 370.096}	260.226	0.522603	newly	97.	1097.19	{9., 2087.}	1083.04	0.801743	Reff	0.392917	0.749785	{0.205593, 1.65497}	106.43	ar	8345.	18 108.8	{15 746., 25 151.}	117.002
8	R0	{2.7904, 17.8966}	{2.68797, 6.07819}	{{2.23722, 0.833992}, {3., 10.1984}}	{12.5344, 66.0372}	inf	456.	1016.74	{51.2039, 3567.31}	167.981	0.358535	newly	172.	960.13	{36., 2857.}	493.68	0.640891	Reff	0.233277	0.651274	{0.16563, 2.237}	191.821	ar	9285.	17 447.5	{14 513., 26 847.}	87.9101
9	R0	{2.51574, 16.376}	{2.60555, 9.16966}	{{1.98859, 5.35572}, {3., 11.9809}}	{19.9141, 44.0055}	inf	368.	116.247	{14.6107, 237.625}	68.4111	0.654646	newly	131.	76.98	{31., 127.}	45.4656	0.33401	Reff	0.296857	0.754745	{0.389574, 1.98839}	154.58	ar	8937.	15 769.7	{14 130., 22 351.}	76.4543
10	R0	{2.56405, 18.1787}	{2.74149, 3.00986}	{{2.23968, 1.55856}, {3., 4.41339}}	{15.5229, 83.4429}	inf	542.	299.809	{34.5218, 425.839}	94.8358	0.745525	newly	185.	681.6	{40., 1451.}	315.73	0.431082	Reff	0.307686	0.64025	{0.126466, 1.53848}	128.435	ar	9005.	16 692.4	{14 821., 21 696.}	85.3684
11	R0	{2.475, 17.1709}	{2.53674, 8.18005}	{{1.71133, 1.66179}, {3., 12.7287}}	{23.8084, 52.361}	inf	441.	160.873	{40.8515, 249.044}	73.939	0.653946	newly	155.	586.28	{22., 704.}	365.729	0.592366	Reff	0.328185	0.504505	{0.190895, 1.26054}	81.2899	ar	8870.	15 248.3	{14 016., 18 601.}	71.9086
12	R0	{2.14974, 11.8619}	{2.79258, 8.92338}	{{2.23178, 3.82199}, {3., 10.4494}}	{29.9032, 24.7727}	inf	247.	495.549	{49.4614, 437.597}	138.463	0.363778	newly	113.	482.67	{26., 941.}	356.699	0.47797	Reff	0.367391	0.602618	{0.106343, 0.744751}	103.119	ar	8414.	16 651.3	{14 990., 22 460.}	97.8993
13	R0	{2.20385, 14.4767}	{2.44533, 11.9757}	{{1.31615, 6.76855}, {3., 16.4935}}	{37.451, 31.7097}	inf	257.	416.31	{271.715, 402.638}	67.4102	0.123042	newly	114.	206.92	{96., 236.}	90.9474	0.215483	Reff	0.397134	0.46316	{0.12926, 0.97411}	55.5133	ar	8347.	15 579.9	{14 486., 18 243.}	86.6524
14	R0	{2.35671, 13.8357}	{2.6766, 4.93909}	{{2.24791, 1.40359}, {3., 8.33262}}	{17.5439, 64.3018}	inf	237.	709.881	{71.7278, 2955.72}	218.568	0.367369	newly	104.	2365.01	{111., 11 555.}	2185.05	0.743699	Reff	0.328997	0.95328	{0.428177, 2.24769}	195.856	ar	8719.	18 261.6	{15 281., 30 260.}	109.446
15	R0	{1.91368, 10.1082}	{1.97535, 5.30894}	{{0.430556, 0.0817529}, {3., 17.6367}}	{64.8965, 75.7836}	inf	135.	615.149	{89.189, 2014.01}	370.094	0.414731	newly	76.	681.8	{32., 1500.}	815.632	0.708133	Reff	0.503681	0.440455	{0.0939244, 0.668481}	38.1998	ar	7486.	16 300.7	{15 027., 19 447.}	117.748
16	R0	{2.48727, 14.882}	{2.76558, 5.4084}	{{2.28964, 1.31517}, {3., 8.50016}}	{16.4336, 63.6581}	inf	327.	504.982	{70.8063, 363.983}	97.4077	0.258089	newly	131.	1516.52	{63., 7711.}	1074.26	0.532054	Reff	0.299467	0.758644	{0.173134, 2.16314}	168.196	ar	8894.	17 025.3	{15 221., 23 970.}	91.4242
17	R0	{2.46795, 13.1915}	{2.59914, 7.38128}	{{1.88043, 0.615411}, {3., 14.9986}}	{21.9147, 56.7694}	inf	235.	758.77	{57.7451, 2112.19}	257.114	0.487836	newly	107.	1674.08	{27., 6950.}	1497.63	0.841537	Reff	0.326757	0.802361	{0.34868, 1.88242}	153.091	ar	8796.	18 638.1	{15 848., 27 724.}	111.892
18	R0	{2.96603, 16.7543}	{2.35271, 4.87295}	{{0.730552, 1.6377}, {3., 7.98206}}	{21.9838, 70.9152}	inf	304.	597.714	{142.621, 545.87}	192.066	0.433431	newly	107.	1078.69	{84., 2875.}	920.944	0.373299	Reff	0.209995	0.535248	{0.0521249, 0.811738}	184.177	ar	9394.	15 924.3	{14 293., 18 254.}	69.5152
19	R0	{2.25216, 9.90465}	{2.44593, 5.78221}	{{1.67838, 0.901307}, {3., 13.7121}}	{29.9144, 70.7439}	inf	114.	891.019	{51.1302, 2974.48}	711.272	0.669745	newly	62.	2316.4	{25., 10 515.}	3669.16	1.0637	Reff	0.321383	0.719899	{0.197089, 1.67916}	134.62	ar	8631.	19 137.6	{16 293., 28 972.}	121.731
20	R0	{2.70837, 18.1923}	{2.73953, 10.7854}	{{2.13175, 7.33246}, {3., 14.2405}}	{13.9244, 40.7142}	inf	470.	297.528	{25.3891, 289.97}	81.6229	0.615533	newly	154.	238.6	{15., 387.}	135.532	0.527457	Reff	0.256212	0.734759	{0.230061, 2.13154}	197.485	ar	9183.	16 457.5	{14 747., 23 384.}	79.2167
21	R0	{2.30288, 15.5877}	{2.51394, 7.84684}	{{1.94336, 0.00971048}, {3., 14.931}}	{23.5282, 49.66}	inf	338.	831.939	{183.361, 2465.23}	176.855	0.366819	newly	137.	1748.59	{80., 7553.}	1194.69	0.674583	Reff	0.344741	0.77688	{0.20842, 1.94316}	140.847	ar	8674.	19 145.3	{15 582., 25 134.}	120.72
22	R0	{2.82208, 10.7544}	{2.4854, 6.39602}	{{1.85862, 4.22128}, {3., 10.099}}	{18.9912, 40.5265}	inf	91.	805.281	{77.3591, 1014.61}	796.166	0.544689	newly	39.	1224.28	{84., 2901.}	3042.46	0.834158	Reff	0.196981	0.734546	{0.0990938, 1.85843}	296.287	ar	9333.	19 615.2	{16 663., 29 109.}	110.17
23	R0	{1.69923, 10.1366}	{2.44565, 4.44063}	{{1.45709, 0.573087}, {3., 7.3591}}	{54.0575, 56.1921}	inf	136.	338.338	{130.98, 324.868}	167.466	0.201034	newly	84.	661.84	{72., 556.}	699.571	0.387804	Reff	0.525912	0.445698	{0.107988, 0.904153}	61.9995	ar	7055.	15 976.	{14 657., 19 468.}	126.449
24	R0	{2.64944, 19.054}	{2.55154, 6.9152}	{{1.98077, 0.0369511}, {3., 14.6724}}	{18.5144, 63.7074}	inf	411.	681.968	{137.473, 2447.64}	129.639	0.382577	newly	150.	1470.05	{75., 5020.}	907.353	0.637434	Reff	0.26097	0.778492	{0.324172, 1.98057}	201.731	ar	9147.	18 348.	{15 354., 24 470.}	100.591
25	R0	{2.53191, 10.9214}	{2.79195, 8.90226}	{{2.22553, 5.30447}, {3., 12.1337}}	{16.0787, 29.8567}	inf	104.	157.049	{104.782, 304.144}	54.7174	0.163417	newly	53.	113.03	{70., 149.}	113.642	0.27595	Reff	0.259774	0.571593	{0.245841, 0.630317}	134.54	ar	9009.	14 872.9	{13 722., 14 646.}	65.0894
26	R0	{1.89155, 6.76944}	{2.57069, 4.69458}	{{1.35901, 0.362719}, {3., 15.2903}}	{46.6802, 103.624}	inf	18.	439.482	{50.7209, 266.748}	2349.17	1.1062	newly	10.	835.05	{27., 716.}	8259.7	1.50343	Reff	0.5058	0.682206	{0.0516667, 1.42347}	76.607	ar	7348.	17 397.3	{15 731., 23 723.}	136.762
27	R0	{2.63149, 15.6979}	{2.78108, 5.76032}	{{2.33659, 1.31054}, {3., 8.48976}}	{13.0807, 63.3052}	inf	374.	295.306	{28.6691, 224.262}	75.2104	0.428059	newly	136.	772.01	{25., 590.}	499.801	0.421904	Reff	0.26236	0.645725	{0.134067, 0.806015}	167.292	ar	9104.	16 013.1	{14 799., 16 222.}	75.891
28	R0	{2.85141, 12.6992}	{2.48232, 3.6771}	{{1.92502, 1.66962}, {3., 12.8569}}	{18.051, 73.2714}	inf	136.	367.362	{9.54168, 130.422}	211.902	0.338373	newly	59.	411.06	{14., 453.}	621.458	0.648442	Reff	0.201595	0.613752	{0.181831, 1.62201}	212.313	ar	9317.	17 417.7	{15 754., 23 720.}	86.9455
29	R0	{2.54244, 12.9085}	{2.75369, 4.61802}	{{1.99257, 1.39209}, {3., 4.5361}}	{17.3724, 68.81}	inf	177.	623.687	{285.655, 851.945}	253.865	0.318157	newly	83.	670.33	{239., 816.}	708.614	0.656935	Reff	0.256278	0.412604	{0.0762378, 0.87498}	98.3263	ar	9034.	16 494.2	{14 794., 19 156.}	82.5795
30	R0	{2.10982, 11.7636}	{2.55924, 10.2074}	{{1.51308, 1.07268}, {3., 18.566}}	{34.3926, 71.9782}	inf	182.	723.729	{29.8769, 1757.14}	321.247	0.562561	newly	78.	928.69	{22., 1141.}	1112.81	0.840549	Reff	0.400444	0.662698	{0.230187, 1.18837}	83.3189	ar	8224.	16 990.6	{14 871., 20 899.}	106.598
31	R0	{2.76312, 15.5396}	{2.42877, 5.07765}	{{1.02868, 3.44068}, {3., 6.5293}}	{24.2739, 67.3244}	inf	308.	192.171	{47.7337, 95.9236}	95.3912	0.585286	newly	111.	260.34	{60., 163.}	187.784	0.267449	Reff	0.234589	0.477905	{0.198667, 0.680226}	116.003	ar	9220.	16 043.7	{15 063., 18 213.}	74.0092
32	R0	{2.21727, 15.5439}	{2.23791, 13.1716}	{{0.892576, 8.20892}, {3., 17.6994}}	{42.8428, 27.2955}	inf	307.	322.618	{25.8462, 1114.85}	103.578	0.511005	newly	111.	175.89	{16., 484.}	131.667	0.471902	Reff	0.413964	0.325622	{0.0107144, 0.896455}	86.9402	ar	8359.	15 413.3	{14 166., 19 010.}	84.392
33	R0	{2.72912, 10.8159}	{1.8538, 12.4061}	{{0.741018, 6.14103}, {3., 19.0583}}	{41.8005, 58.789}	inf	49.	305.629	{94.0383, 799.148}	527.141	0.590914	newly	25.	175.85	{30., 479.}	608.76	0.606349	Reff	0.222969	0.33711	{0.0775071, 0.942192}	105.253	ar	9196.	18 221.	{16 558., 23 709.}	98.1406
34	R0	{2.20928, 10.1308}	{2.60008, 4.63287}	{{1.94758, 4.12609}, {3., 4.76758}}	{26.6916, 54.2695}	inf	59.	423.354	{162.691, 276.938}	620.206	0.525059	newly	37.	611.13	{128., 312.}	1552.3	0.764142	Reff	0.360775	0.622442	{0.0997199, 1.94738}	101.935	ar	8388.	18 352.7	{16 349., 25 175.}	118.797
35	R0	{2.10103, 15.2673}	{2.51753, 3.91296}	{{1.6191, 0.320484}, {3., 6.39377}}	{35.7996, 74.3703}	inf	355.	430.226	{204.234, 204.234}	104.471	0.338584	newly	143.	590.19	{143., 521.}	319.839	0.310414	Reff	0.409701	0.398476	{0.130526, 0.783223}	63.9227	ar	8264.	16 111.6	{15 058., 16 924.}	94.9608
36	R0	{2.74914, 19.2775}	{2.64163, 6.78618}	{{1.94596, 0.395222}, {3., 10.3949}}	{15.9559, 64.7974}	inf	508.	950.95	{138.408, 2882.83}	124.025	0.329099	newly	161.	2005.4	{79., 8130.}	1161.14	0.724819	Reff	0.249622	0.852167	{0.363661, 1.94577}	242.471	ar	9301.	19 226.4	{15 560., 26 459.}	106.714
37	R0	{2.78161, 16.0136}	{2.56509, 2.8727}	{{2.07464, 1.34606}, {3., 4.07006}}	{16.2631, 82.0609}	inf	295.	331.39	{26.7631, 545.679}	106.321	0.498973	newly	117.	876.93	{61., 2444.}	666.385	0.570825	Reff	0.232264	0.87952	{0.338482, 2.07444}	281.453	ar	9240.	17 529.4	{14 852., 24 874.}	89.7121
38	R0	{2.4454, 8.33501}	{2.40657, 4.64158}	{{0.668931, 0.707448}, {3., 13.8043}}	{27.896, 83.6719}	inf	27.	281.957	{51.4656, 192.888}	948.55	0.807366	newly	18.	745.64	{20., 533.}	4048.67	1.12751	Reff	0.282933	0.615285	{0.135838, 1.85123}	139.508	ar	8853.	17 635.7	{16 268., 21 791.}	99.2058
39	R0	{2.9154, 12.3861}	{2.38282, 6.75491}	{{1.37334, 1.06042}, {3., 14.3002}}	{21.8661, 58.6641}	inf	88.	848.082	{37.0016, 1066.43}	880.427	0.758844	newly	42.	1382.73	{22., 4368.}	3207.36	1.07191	Reff	0.190376	0.602681	{0.0461869, 1.37313}	262.748	ar	9371.	18 905.2	{16 651., 24 152.}	101.741
40	R0	{2.40923, 15.1963}	{2.64473, 8.39033}	{{1.98494, 5.3678}, {3., 11.8364}}	{22.1026, 44.787}	inf	294.	86.1881	{57.3233, 181.07}	70.6843	0.644471	newly	115.	61.5	{29., 99.}	48.7826	0.370203	Reff	0.339701	0.581753	{0.257194, 0.970418}	86.5661	ar	8735.	14 854.3	{14 174., 14 967.}	70.0544
41	R0	{2.63137, 15.238}	{2.5614, 10.218}	{{1.89855, 3.07604}, {3., 17.6405}}	{21.9916, 48.3078}	inf	281.	921.138	{72.5982, 1329.17}	260.497	0.486922	newly	89.	1132.11	{45., 2383.}	1189.97	0.744158	Reff	0.24998	0.743106	{0.459543, 1.59931}	212.263	ar	9150.	17 427.5	{14 940., 23 872.}	90.4647
42	R0	{2.48769, 19.4593}	{2.17197, 12.3951}	{{0.607845, 7.36289}, {3., 18.9711}}	{36.1684, 36.3024}	inf	467.	322.886	{208.347, 778.233}	60.1234	0.352213	newly	165.	157.3	{63., 257.}	42.2667	0.213068	Reff	0.312454	0.404343	{0.0706585, 0.59803}	66.4771	ar	8932.	16 043.1	{14 644., 19 505.}	79.6133
43	R0	{2.61199, 10.214}	{2.54421, 6.34262}	{{1.9444, 4.12949}, {3., 10.2771}}	{18.6384, 38.3473}	inf	68.	552.274	{43.2201, 348.817}	725.794	0.62562	newly	40.	866.31	{62., 1071.}	2066.73	0.785039	Reff	0.251796	0.835046	{0.120421, 1.9442}	251.897	ar	9061.	19 977.5	{16 905., 27 985.}	120.478
44	R0	{2.50994, 18.3382}	{2.47006, 5.99337}	{{1.45163, 1.62312}, {3., 11.8151}}	{24.2378, 67.3176}	inf	483.	891.734	{112.899, 2221.08}	123.127	0.286649	newly	173.	1326.16	{71., 5498.}	687.295	0.585444	Reff	0.350137	0.573559	{0.24811, 0.882061}	76.7574	ar	8827.	16 365.2	{14 307., 21 450.}	85.3988
45	R0	{2.44283, 16.8338}	{2.57236, 3.75981}	{{1.88948, 0.43698}, {3., 6.34233}}	{23.8921, 77.6651}	inf	399.	763.199	{264.505, 683.101}	153.133	0.296443	newly	142.	969.74	{200., 4152.}	586.155	0.491455	Reff	0.308285	0.540304	{0.144171, 1.0517}	100.677	ar	8878.	17 402.6	{15 490., 23 873.}	96.0196
46	R0	{2.51815, 18.1462}	{2.63335, 7.03793}	{{1.98152, 0.0935203}, {3., 11.8776}}	{19.9182, 61.2154}	inf	537.	792.197	{234.207, 1468.45}	78.5681	0.206623	newly	173.	1417.68	{145., 2906.}	727.514	0.595861	Reff	0.31905	0.821635	{0.355679, 1.98132}	163.711	ar	8978.	18 378.8	{15 780., 25 794.}	104.71
47	R0	{2.88885, 16.8987}	{2.63211, 10.8903}	{{1.76297, 1.02475}, {3., 19.315}}	{14.1971, 48.7318}	inf	414.	853.456	{161.566, 1628.02}	131.363	0.336771	newly	129.	1039.52	{57., 1540.}	725.178	0.693938	Reff	0.22533	0.753385	{0.281574, 1.58098}	241.206	ar	9337.	17 475.5	{15 231., 22 887.}	87.1639
48	R0	{2.58709, 13.8786}	{2.1976, 5.02268}	{{0.886691, 3.51299}, {3., 6.594}}	{34.846, 63.8099}	inf	256.	257.921	{112.431, 594.271}	84.0297	0.390256	newly	109.	316.6	{74., 759.}	218.128	0.331662	Reff	0.243704	0.417602	{0.0880154, 0.653002}	102.821	ar	9146.	15 733.5	{14 867., 17 742.}	72.0262
49	R0	{2.42959, 10.7132}	{2.35423, 6.09814}	{{1.67912, 1.0269}, {3., 13.3795}}	{27.0488, 63.367}	inf	116.	556.86	{46.6619, 894.271}	407.368	0.60266	newly	60.	1311.43	{21., 3367.}	2114.45	0.963685	Reff	0.288149	0.617875	{0.0812928, 1.43912}	131.424	ar	8863.	18 211.4	{16 426., 24 471.}	105.477
50	R0	{2.60513, 13.2796}	{1.91365, 3.14548}	{{0.134237, 2.44824}, {3., 3.47728}}	{45.338, 77.1285}	inf	243.	623.778	{84.5018, 1679.47}	179.428	0.363624	newly	108.	708.68	{89., 2069.}	563.5	0.651172	Reff	0.256605	0.336109	{0.0174191, 0.638025}	94.5064	ar	9095.	16 512.3	{14 757., 19 098.}	81.5539
